# Anemia in pregnancy and sleep of 6-month-old infants: A prospective cohort study

**DOI:** 10.3389/fnut.2023.1049219

**Published:** 2023-03-10

**Authors:** Lei Zhang, Shuangshuang Ma, Feicai Dai, Qiong Li, Lin Wu, Lijun Yu, Tianqin Xie, Dao-min Zhu, Peng Zhu

**Affiliations:** ^1^Department of Maternal, Child & Adolescent Health, School of Public Health, Anhui Medical University, Hefei, China; ^2^MOE Key Laboratory of Population Health Across Life Cycle, Hefei, China; ^3^NHC Key Laboratory of Study on Abnormal Gametes and Reproductive Tract, Hefei, China; ^4^Anhui Provincial Key Laboratory of Population Health and Aristogenics, Hefei, China; ^5^Department of Sleep Disorders, Affiliated Psychological Hospital of Anhui Medical University, Hefei, China; ^6^Hefei Fourth People’s Hospital, Hefei, China; ^7^Anhui Mental Health Center, Hefei, China

**Keywords:** anemia in pregnancy, infant sleep, hemoglobin, iron supplementation, cohort study

## Abstract

**Objective:**

Anemia has been reported to adversely influence sleep in infants. However, the association between anemia in pregnancy and infant sleep remains unclear. We aimed to examine the association between maternal anemia in pregnancy and sleep parameters of 6-month-old infants.

**Methods:**

We enrolled 2,410 mother-infant pairs between 2018 and 2021 in Hefei. Data on maternal hemoglobin concentration were collected at 24–28 gestational weeks from the electronic medical records of the hospitals. Nocturnal and daytime sleep duration, number of night awakenings, nocturnal wakefulness, and sleep latency of infants aged 6 months were measured using the Brief Infant Sleep Questionnaire with five items. A restricted cubic spline model was used to examine the relationship between maternal hemoglobin concentration and infant nocturnal sleep duration after adjusting for potential confounders.

**Results:**

In our study, 807 (33.5%) mothers had anemia during pregnancy. Compared to infants born to mothers without anemia, infants born to mothers with anemia in pregnancy had shorter nocturnal sleep duration [mean (SD), 560.29 (79.57) mins vs. 574.27 (75.36) mins] at the age of 6 months. Subgroup analysis showed consistent significant differences in nocturnal sleep duration between infant born to anemic and non-anemic mothers, except in case of stratification by preterm birth [mean difference (mins), 2.03 (95% CI, −20.01, −24.07)] and pre-pregnancy obesity [mean difference (mins), −0.85 (95% CI, −16.86, −15.16)]. A J-shaped nonlinear correlation curve was observed between maternal hemoglobin concentration and infant nocturnal sleep duration. Compared with mothers without daily iron supplementation, mothers who had daily iron supplementation had higher hemoglobin concentrations [mean (SD), 112.39 (11.33) g/L vs. 110.66 (10.65) g/L] at delivery and their infants had longer nocturnal sleep duration [mean (SD), 565.99 (82.46) mins vs. 553.66 (76.03) mins].

**Conclusion:**

Anemia in pregnancy may have an adverse influence on the sleep of 6-mon-old infants, and the relationship between maternal hemoglobin concentration and nocturnal sleep duration is nonlinear.

## 1. Introduction

Anemia in pregnancy, characterized by hemoglobin (Hb) level of < 110 g/l, is a complication caused by maternal malnutrition, it is one of the most critical health conditions and an urgent public health priority worldwide (1, 2). A recent meta-analysis of the global prevalence of anemia in pregnant women indicated that the overall prevalence of anemia in pregnancy was 36.8% (3). There is broad agreement that anemia in pregnancy is associated with adverse fetal growth and infant development outcomes, such as fetal malformation, low birth weight, and autism spectrum disorder (4–6). It is worth noting that sleep disorders seem to be more common in infants with developmental problems, especially in the first few years of life. Additionally, development of infant circadian system begins *in utero* and continues throughout early-life; a damaged intrauterine environment, as a consequence of low Hb concentration, may disrupt infant sleep development *in utero* and after birth (7). Therefore, anemia in pregnancy may have a potential correlation with infant sleep. Previous studies have demonstrated associations between infant anemia and sleep, such as associations between wakefulness duration and sleep state, during infancy (8, 9). However, investigations on the associations of maternal anemia in pregnancy with infant sleep are still limited.

Sleep development is an active, complex neurophysiological process, and infant sleep development has specific time sequences, similar to other organs or functions (10). The sixth month is a key time point of the sleep development process, and trends of nocturnal sleep duration (NSD), defined as 19:00–7:00, showed rapid changes over the first 6 months before stabilizing to a plateau (11). After birth, NSD gradually increases and daytime sleep duration (DSD) gradually decreases, eventually leading to a stable sleep pattern, but there is still no accepted recommended range for NSD, DSD, or other sleep parameters. Previous studies on sleep in 6-month-old infants revealed that NSD was 491–572 min and DSD was 186–272 min (12, 13). In addition, half of the infants achieved self-regulated sleep at 6 months of age, and their electrical patterns of non-rapid eye movement and rapid eye movement (REM) sleep progressively resembled those seen in adults (14). Changes in the intrauterine environment caused by anemia, such as chronic intrauterine hypoxia, may impair the development of infant sleep. Sleep is interlinked throughout the lifespan, and poor sleep at 6 months of age increases the risk of sleep disorders later in life (15). Moreover, previous studies have suggested that children with higher proportions of sleep at night perform better on executive functions, whereas children with shorter NSDs are at higher risk of obesity and physical inactivity (16, 17). Therefore, understanding the relationship between maternal anemia in pregnancy and infant sleep at 6 months of age is critical for developing preventive strategies to promote healthy infant development.

In this prospective cohort study, we aimed to determine the potential impact of maternal anemia in pregnancy on infant sleep parameters at 6 months of age. We further examined the nonlinear relationship between maternal Hb concentration and infant NSD using a restricted cubic spline model.

## 2. Methods

### 2.1. Participants and study design

This was a prospective birth cohort study. From March 2018 to June 2021, 4,216 pregnant women at 16–23 gestational weeks were recruited from three hospitals including the Hefei First People’s Hospital, the Hefei Maternal and Child Care Hospital, and the First Affiliated Hospital of Anhui Medical University. The inclusion criteria for this study included the following: pregnant women, aged 18–45 years, 16–23 gestational weeks, no communication difficulties and lived in Hefei, single gestation, and pregnancy without assisted reproductive technology.

Exclusion criteria were the following: missing maternal blood samples, stillbirth, and birth defects. Finally, our study included 2,410 mother-infant pairs with complete data (Supplemental Figure 1). Ethics committee approval for the study was obtained from the ethics committee of Anhui Medical University (No. 20180092), and all patients provided written informed consent before participating in the study.

During recruitment, pregnant women were required to complete structured questionnaires regarding demographic characteristics, food frequency intake, health status, and iron supplementation. After recruitment, we obtained maternal Hb concentration data from electronic medical records of hospitals at 24–28 gestational weeks. At delivery, information on maternal Hb concentration at delivery and newborn birth outcomes was collected from electronic medical records. In the sixth month after delivery, we assessed the infants’ sleep; infants’ sleep parameters, at 6 months of age, were obtained from their parents *via* a questionnaire.

### 2.2. Outcome measures

#### 2.2.1. Secondary data

The diagnosis of anemia in pregnancy was based on the Hb concentration according to the WHO standard (18). Hb < 110 g/l was defined as anemia, and pregnant women were classified into two groups according to Hb concentration (Hb < 110 g/l, and Hb ≥ 110 g/l). Maternal Hb concentration was measured using an Auto Hematology Analyzer BC6800plus (Shenzhen Mindray Bio-Medical Electronics Co., Ltd., China) using venous blood samples from the hospitals.

#### 2.2.2. Measurement of infants’ sleep parameters

Data on infants’ sleep parameters were collected at 6 months of age. Infant sleep was assessed using a face-to-face survey based on the Brief Infant Sleep Questionnaire (BISQ). The BISQ includes 11 questions on daytime and nocturnal sleep patterns and behaviors and is widely used in the assessment of infant sleep parameters (19). The validity and reliability of the BISQ (*r* = 0.82–0.95) have been widely reported (20). During the investigation, mothers completed the items of the translated BISQ concerning the infants’ sleep parameters. The following five items were used to calculate the infants’ sleep parameters: “How long does your baby sleep at night (19:00–7:00),” “How long does your baby sleep in the daytime (7:00–19:00),” “How long does your baby awake at night,” “How long does your baby need to fall asleep,” as well as “How many times your baby wakes up each night on average?”

### 2.3. Measurement of potential confounders

The characteristics of mothers during pregnancy were collected through face-to-face interviews, including maternal age at delivery, years of education > 12 years, family income < 10,000 yuan/month, and pre-pregnancy BMI. Maternal systolic blood pressure (SBP) and diastolic blood pressure (DBP) were obtained from the electronic medical records. The past month’s maternal diet (fruit, dessert, vegetable, and bean product intake) was assessed using an adapted food frequency questionnaire (21). With responses ranging from “never,” “one to two times a week,” “three to six times a week” to “1 time a day or more.” The characteristics of infants were collected from electronic medical records, including gestational age at birth, birth weight and gender, preterm birth, small for gestational age (SGA), and large for gestational age (LGA). LGA (birth weight > 90th percentile of newborns at the same gestational age) and SGA (birth weight < 10th percentile of newborns at the same gestational age) were defined as the gestational age- and sex-specific international reference for fetal growth (22). Breastfeeding was defined as receiving breast milk and no other food or drink during the first 6 months after birth. In the end, the following potential confounders were adjusted in the models due to the previous literature and the effect on anemia and NSD, include maternal age at delivery, education levels, family income, pre-pregnancy BMI (23), SBP, DBP, maternal diet (fruits, dessert, vegetables, bean products) (24), infant gender, preterm birth, SGA, LGA, breastfeeding (25).

### 2.4. Statistical analysis

The Shapiro–Wilk test was used to confirm the normality of the distribution of variables. Continuous variables with a normal distribution are expressed as mean (SD), and categorical variables are expressed as numbers (percentages). For variables with non-normal distribution, the results are expressed as medians (interquartile range). Characteristics of mothers and infants were compared between mothers with anemia and mothers without anemia using Student’s *t*-test for continuous variables and Chi-square analysis for categorical variables. Comparison of sleep parameters between infants born to mothers with anemia and mothers without anemia was performed using covariance analysis. We further conducted subgroup analyses for the primary outcome stratified by mother (education level and family income) and infants characteristics (gender, preterm birth, SGA, LGA, and breastfeeding). In addition, a nonlinear model was fitted with restricted cubic spline curves to examine the nonlinear association between maternal Hb and NSD in 6-month-old infants after adjusting for potential confounders. We also examined the difference in infant sleep between mothers with anemia and without daily iron supplementation using covariance analysis. All statistical analyses were performed using the SPSS statistical software (SPSS Statistics 21.0; SPSS Inc.) and R (version 4.0.2, R Foundation for Statistical Computing). *p* < 0.05 was considered statistically significant, and all tests were two-sided.

## 3. Results

### 3.1. Characteristics of the study population

The characteristics of 2,410 mother-infant pairs are presented in total and by maternal anemia in Table 1. Of the mothers, 807(33.5%) had anemia during pregnancy, 1,618 (67.1%) had received education for more than 12 years, and 1,262 (52.4%) had a family income lower than 10,000 yuan every month. The mean (SD) age at delivery was 30.93 (4.29) years, and the mean (SD) pre-pregnancy BMI was 21.46 (2.92) kg/m^2^. Of the infants included in the study, 1,258 (52.2%) were male, 1,152 (47.8%) were female, 225 (9.3%) were preterm birth, and 1,354 (56.2%) were breastfeeding. Compared with mothers without anemia, mothers with anemia in pregnancy had lower pre-BMI [mean (SD), 21.17 (2.60) kg/m^2^ vs. 21.61 (3.06) kg/m^2^], SBP [mean (SD), 108.62 (9.58) mmHg vs. 111.61 (10.22) mmHg], and DBP [mean (SD), 67.19 (7.08) mmHg vs. 70.04 (7.66) mmHg]. Compared with children born to mothers without anemia, those born to mothers with anemia had a higher birth weight [mean (SD), 3.49 (0.59) kg vs. 3.43 (0.58) kg] and lower SGA percentage (6.7 vs. 9.5%).

**Table 1:** Characteristics of the 2,410 mother–child pairs in this analysis.

Variables	Total (*n* = 2,410)	Anemia (*n* = 807)	Non-anemia (*n* = 1,603)	*p* Value^a^
Maternal characteristics				
Age at delivery, years, mean (± SD)	30.93 ± 4.29	31.09 ± 4.29	30.8 ± 4.29	0.190
Educational years > 12 years, *n* (%)	1,618(67.1)	525(65.1)	1,093(68.2)	0.123
Family income < 10,000 yuan/mon, *n* (%)	1,262(52.4)	429(53.2)	833(52.0)	0.579
Pre-pregnancy BMI, Kg/m^2^, mean (± SD)	21.46(2.92)	21.17 ± 2.6	21.61 ± 3.06	<0.001
SBP, mmHg, mean (± SD)	110.60(10.11)	108.62 ± 9.58	111.61 ± 10.22	<0.001
DBP, mmHg, mean (± SD)	69.08(7.59)	67.19 ± 7.08	70.04 ± 7.66	<0.001
Daily iron supplement, *n* (%)	801(33.2)	434(53.8)	367(22.9)	<0.001
Fruits frequency ≥ 3 time/week, *n* (%)	2,270(94.2)	761(94.3)	1,509(94.1)	0.963
Dessert frequency ≥ 3 time/week, *n* (%)	420(17.4)	147(18.2)	273(17.0)	0.477
Vegetables frequency ≥ 3 time/week, *n* (%)	2,335(96.9)	783(97.0)	1,552(96.8)	0.907
Bean products frequency ≥ 3 time/week, *n* (%)	1,189(49.3)	377(46.7)	812(50.7)	0.064
Infant characteristics				
Male, *n* (%)	1,258(52.2)	409(50.7)	849(53.0)	0.290
Gestational age at birth, week, mean (± SD)	39.03(1.37)	38.98 ± 2.15	39.01 ± 1.75	0.704
Birth weight, Kg, mean (± SD)	3.41(0.46)	3.49 ± 0.59	3.43 ± 0.58	0.016
Preterm birth, *n* (%)	225(9.3)	75(9.3)	150(9.4)	0.959
SGA, *n* (%)	207(8.6)	54(6.7)	153(9.5)	0.018
LGA, *n* (%)	335(13.9)	117(14.5)	218(13.6)	0.547
Breastfeeding, *n* (%)	1,354(56.2)	465(57.6)	889(55.5)	0.313

^a^*p* Value of Student *t*-test or Chi-square test.

BMI: body mass index; SBP: systolic blood pressure; DBP: diastolic blood pressure; SGA: small for gestational age; LGA: large for gestational age.

### 3.2. Nonlinear association between maternal Hb concentration and infant NSD

**Figure 1:**
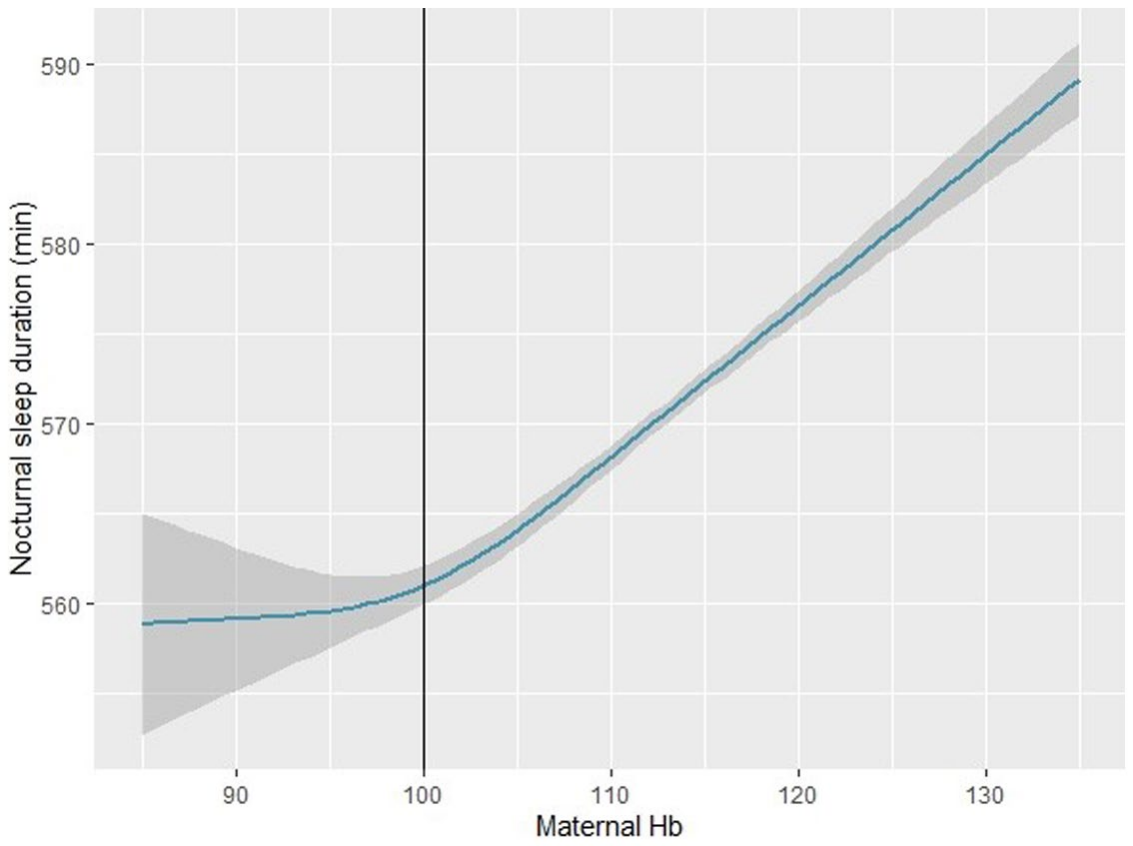
Association between maternal hemoglobin concentration and infant nocturnal sleep duration in the restricted cubic spline model (*n* = 2,410). The restricted cubic spline model adjusted for maternal age at delivery, education levels, family income, pre-pregnancy BMI, SBP, DBP, daily iron supplementation, maternal diet (fruits, dessert, vegetables, bean products), infant gender, preterm birth, SGA, LGA, breastfeeding. SGA: small for gestational age; LGA: large for gestational age. BMI: Body mass index; SBP: systolic blood pressure; DBP: diastolic blood pressure; SGA: small for gestational age; LGA: large for gestational age.

Using a nonlinear regression model, we found that the association between maternal Hb concentration and infant NSD was J-shaped (nonlinear *p* < 0.001) after adjusting for confounders (Figure 1). A flattened increase in NSD was observed when maternal Hb concentration was < 100 g/l, whereas a dramatical increase in NSD was observed when maternal Hb concentration was > 100 g/l.

### 3.3. Comparison of sleep parameters between infants born to mothers with anemia vs. those born to mothers without anemia

We compared six types of infant sleep parameters between the two groups, including NSD, daytime sleep duration, number of night awakenings, nocturnal wakefulness, sleep latency, and total sleep duration (Table 2). Infants born to mothers with anemia had significantly shorter NSD than infants born to mothers without anemia [mean (SD), 560.29 (79.57) mins vs. 574.27 (75.36) mins]. However, there were no significant differences in the other five infant sleep parameters between the two groups.

**Table 2:** Comparison of sleep parameters between infants born to mothers with anemia vs. mothers without anemia (*n* = 2,410).

Sleep parameters^*^	Total (*n* = 2,410)	Anemia (*n* = 807)	Non-anemia (*n* = 1,603)	*p* Value^a^
Nocturnal sleep duration (min)	569.59(77.06)	560.29(79.57)	574.27(75.36)	<0.001
Daytime sleep duration (min)	233.22(93.24)	237.59(97.78)	231.02(90.82)	0.265
Number of night awakening (*n*)	2.00(2.00)	2.00(2.00)	2.00(2.00)	0.954
Nocturnal wakefulness (min)	30.00(40.00)	30.00(40.00)	30.00(40.00)	0.837
Sleep latency (min)	30.00(10.00)	30.00(10.00)	30.00(10.00)	0.922
Total sleep duration (min)	802.45(117.37)	798.40(120.25)	804.48(115.88)	0.114

^*^Mean (SD) or median (IQR).

^a^*p* Value of covariance analysis. The covariance analysis model adjusted for maternal age at delivery, education levels, family income, pre-pregnancy BMI, SBP, DBP, daily iron supplementation, maternal diet (fruits, dessert, vegetables, bean products), infant gender, preterm birth, SGA, LGA, breastfeeding.

SGA: small for gestational age; LGA: large for gestational age. BMI: Body mass index; SBP: systolic blood pressure; DBP: diastolic blood pressure; SGA: small for gestational age; LGA: large for gestational age.

### 3.4. Subgroup analyses on differences in infant NSD

**Figure 2:**
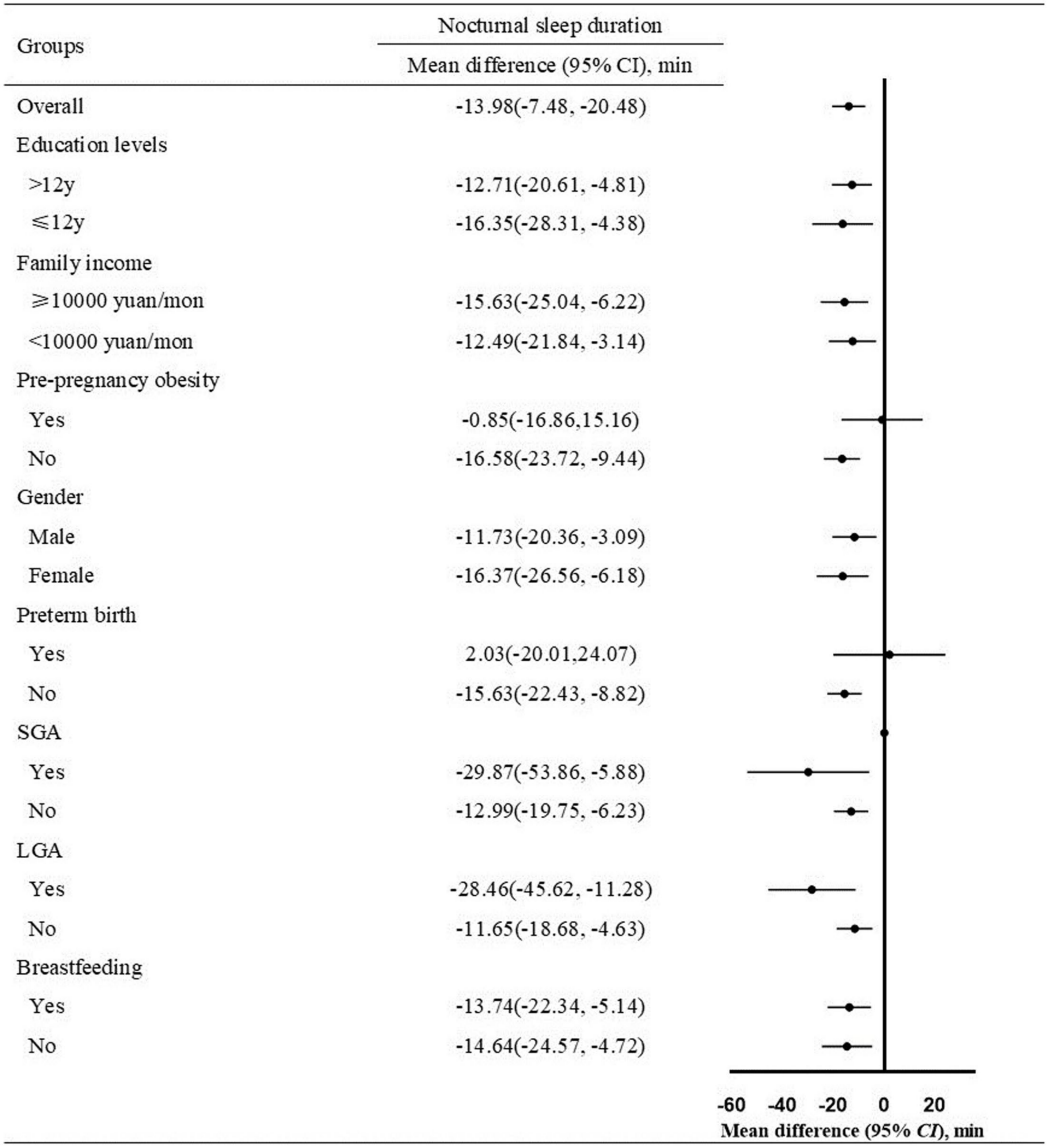
Mean difference of nocturnal sleep duration in infants born to mothers with anemia vs. mothers without anemia (*n* = 1,347, Mean difference ± SD). Infants of the two groups were divided into subgroups according to maternal education levels, family income, pre-pregnancy obesity, infant gender, preterm birth, SGA, LGA, breastfeeding. The covariance analysis model adjusted for maternal age at delivery, education levels (if not stratified), family income (if not stratified), pre-pregnancy BMI (if not stratified), SBP, DBP, daily iron supplementation, maternal diet (fruits, dessert, vegetables, and bean products), infant gender, preterm birth, SGA (if not stratified), LGA (if not stratified), breastfeeding (if not stratified). SGA: small for gestational age; LGA: large for gestational age. BMI: body mass index; SBP: systolic blood pressure; DBP: diastolic blood pressure; SGA: small for gestational age; LGA: large for gestational age.

We examined whether the associations differed by potential effect modifiers including maternal education level (> 12 years, ≤ 12 years), family income (≥ 10,000 yuan/month, < 10,000 yuan/month), infant gender (male, female), preterm birth (yes, no), SGA (yes, no), LGA (yes, no), and breastfeeding (yes, no) in the subgroup analysis (Supplementary Table 1). Compared with infants born to mothers without anemia, those born to mothers with anemia had a 13.98 min(95% CI, 7.48–20.48) shorter NSD (Figure 2). After grouping by maternal education level, family income, infant gender, SGA, LGA, and breastfeeding, infants born to mothers with anemia had a shorter NSD than infants born to mothers without anemia, in all subgroups. However, the difference in NSD did not exist in infants with preterm birth [mean difference (min), 2.03 (95% CI, −20.01, –24.07)], and infants born to mothers with pre-pregnancy obesity [mean difference (min), −0.85 (95% CI, −16.86, –15.16)].

### 3.5. Comparison of maternal Hb concentration and infant sleep parameters between anemic mothers with daily iron supplementation vs. those without daily iron supplementation

We compared maternal Hb concentration and infant sleep parameters between the two groups and found that daily iron supplementation during pregnancy had a positive influence on maternal Hb concentration and infant sleep parameters. We found that mothers in the daily iron supplementation group had higher Hb concentrations [mean (SD): 112.39 (11.33) g/L vs. 110.66 (10.65) g/L] at delivery (Table 3). Infants in the daily iron supple-mentation group had significantly longer NSD than those without the daily iron supplementation group [mean (SD), 565.99 (82.46) mins vs. 553.66 (76.03) mins]. However, we did not find any significant difference in other infant sleep parameters.

**Table 3:** Comparison of maternal Hb concentration and infant sleep parameters in anemic mothers with daily iron supplementation vs. mothers without daily iron supplementation (*n* = 807).

Maternal Hb concentration and infant sleep parameters^*^	Daily iron supplementation (*n* = 434)	Non-daily iron supplementation (*n* = 373)	*p* Value^a^
Hb concentration in the second trimester (g/L)	103.11(6.30)	103.61(4.91)	0.214
Hb concentration at delivery (g/L)	112.39(11.33)	110.66(10.65)	0.039^#^
Nocturnal sleep duration (min)	565.99(82.46)	553.66(76.03)	0.028^#^
Daytime sleep duration (min)	235.23(95.64)	240.34(99.34)	0.458
Number of night awakenings (*n*)	2.00(2.00)	2.00(2.00)	0.910
Nocturnal wakefulness (min)	30.00(40.00)	30.00(40.00)	0.753
Sleep latency (min)	30.00(10.00)	30.00(10.00)	0.902
Total sleep duration (min)	801.02(122.69)	793.84(117.42)	0.398

^*^Mean (SD) or median (IQR);

^#^*p* < 0.05;

^a^*p* Value of covariance analysis. The covariance analysis model of infant sleep parameters adjusted for maternal age at delivery, education levels, family income, pre-pregnancy BMI, SBP, DBP, maternal diet (fruits, dessert, vegetables, bean products), infant gender, preterm birth, SGA, LGA, breastfeeding.

SGA: small for gestational age; LGA: large for gestational age. BMI: Body mass index; SBP: systolic blood pressure; DBP: diastolic blood pressure; SGA: small for gestational age; LGA: large for gestational age.

## 4. Discussion

Our study adds to the scarce literature on the association between anemia in pregnancy and infant sleep. In this prospective cohort study, we also observed a J-shaped nonlinear association between maternal Hb concentration and infant NSD. We found that infants born to mothers with anemia in pregnancy had shorter NSD at 6 months of age, and the differences were consistent across subgroups except in preterm birth infants and infants born to mothers with pre-pregnancy obesity. Daily iron supplementation may have a positive influence on maternal anemia and infant NSD.

Consistent with our study, a study conducted in Japan investigated a nonlinear relationship between maternal Hb concentration and infant sleep, and a U-shaped correlation curve between maternal Hb level and the risk of infant sleep at 22:00 or later was reported (23). The nonlinear relationship between maternal Hb concentration and infant sleep might be a possible cause for the conflicting findings reported in literature. We observed a J-shaped nonlinear correlation curve between maternal Hb concentration and infant NSD. The underlying mechanism of this nonlinear association is unclear, but we speculate that it may be related to health behaviors and medical adherence in pregnant women. When mothers have moderate or severe anemia, they are more likely to adopt a healthier lifestyle or stricter medical practices to ensure that their fetus receives adequate nutrition. These changes may attenuate the adverse effects of anemia to some extent. In our study, infants born to mothers with anemia (especially those with Hb concentration of 90–110 g/l) had a shorter NSD than infants born to mothers without anemia. It is noteworthy that 97.7% of anemia in pregnancy is mild anemia (Hb concentration 100–109 g/l) or moderate anemia (Hb concentration 90–99 g/l) worldwide (3). Therefore, it might be a tremendous opportunity to improve infant early-life sleep development and decrease the risk of numerous disorders originating from sleep by providing timely interventions for anemia in pregnancy. Furthermore, follow-up studies are needed to investigate the effect of interventions toward maternal anemia on infants’ sleep development.

Our findings suggest that anemia in pregnancy may be associated with shorter NSD in infants. In line with our study, a study in Chile suggested that iron deficiency anemia (IDA) is associated with altered infants’ sleep states; children with IDA in infancy showed shorter REM sleep episodes in the last third (9). Similar negative association between Hb levels and sleep durations was also found in the English Longitudinal Study of Ageing (26). Contrary to our findings, a study in Nepal and Zanzibar reported that IDA infants slept longer at night than non-IDA infants (27); however, as it was an intervention study, the effects on sleep could have been confounded by effects of administering iron or folic acid supplements. In contrast, iron deficiency has been reported to only account for 75% of maternal anemia (28), and the association between anemia and sleep may be moderated. Therefore, there is an urgent need to conduct more studies with sufficient samples, to determine the effect of anemia in pregnancy on infant sleep development.

Notably, NSD did not differ between two groups when the infants in both had preterm birth or had mothers with pre-pregnancy obesity. One reason may be that the sample sizes of preterm birth infants and mothers with pre-pregnancy obesity were too small, which may have increased the risk of type II errors. Furthermore, recent studies have shown that maternal pre-pregnancy obesity and preterm birth are closely corelated with infant neurodevelopment, and this association may have confounding effects on assessment of the real-world association between maternal anemia and infant sleep. Our results showed that infants whose mothers had daily iron supplementation exhibited a longer NSD. Iron supplementation has been recommended for health care during pregnancy and helps prevent and treat anemia in pregnancy. The severity of fatigue symptoms, daytime sleepiness, sleep quality, and sleep-related breathing disorders are mitigated after iron supplementation in non-pregnant participants with anemia (29, 30). However, evidence of the potential impact of iron supplementation during pregnancy on early infant sleep is limited. Our results suggest that daily iron supplementation in pregnancy may optimize the NSD in infants.

Several possible mechanisms may explain the association between maternal anemia and infant sleep. Anemia can cause reduced peripheral oxygen saturation or hypoxia, both of which are key drivers of hypothalamic–pituitary–adrenal (HPA) axis alterations (31, 32). Animal experiments also elucidated that intermittent hypoxia during gestation results in differential alterations in the HPA axis and behavior of the offspring (33). Therefore, anemia in pregnancy may affect infants’ HPA axis function and lead to sleep problems. Iron deficiency is considered to be one of the most important risk factors for infant sleep disorders. Animal studies have shown that the hippocampus is very sensitive to a lack of iron during early development (34), and iron deficiency during pregnancy may damage fetal hippocampal development and lead to sleep disorders in infancy. Although numerous studies on human have been carried out, findings regarding the relationship between maternal iron deficiency and infant sleep have been inconsistent (8, 35). One reason may be that iron supplementation, which is universally used to prevent anemia during pregnancy, alleviates the negative effects of iron deficiency. Perinatal complications and infant anemia caused by anemia are also considered to be possible mechanisms between anemia in pregnancy and infant sleep (36–38). Further well-designed studies are required to understand these mechanisms.

Our study has several advantages. First, this is the first study to report nonlinear associations between maternal Hb concentration and NSD in 6-month-old infants. Second, the prospective study design revealed clear time-ordered relationships between maternal anemia and infant sleep. Third, statistical analyses were performed using multiple covariates. Our study has some limitations. Information on infants’ sleep parameters has been reported by parents, and recall bias may exist. Objective measures, such as actigraphy and polysomnography readings, could be used in future studies. Performing actigraphy is not feasible sometimes due to logistical or financial reasons, while the BISQ has been validated against actigraphy and is widely used in infant sleep measurement (39). Second, this was an observational study, and thus, we could not establish a causal relationship between anemia and infant sleep. Third, we did not collect the data on infant Hb concentration. The close association of infant anemia with maternal anemia and infant sleep might be an important mediator in this study. Therefore, we could not eliminate interference of such confounding factors from infant anemia, and caution is required when interpreting the results of this study. Last, we collect maternal Hb concentrations at 24–28 gestational weeks and at delivery, and maternal anemia condition during the whole pregnancy was not clear. Future studies should collect Hb data at more time points to evaluate the effects of anemia duration and period on infant sleep.

## 5. Conclusion

In this prospective birth cohort study, we found that maternal anemia in pregnancy was associated with shorter NSD in infants aged 6 months. The relationship between maternal Hb concentration and infant NSD was nonlinear. Daily iron supplementation may have a positive influence on maternal anemia and infant NSD. Our study highlights the dangers of anemia in pregnancy and the potential benefits of preventing it on infant sleep. In the future, clinical medical facilities should include prevention, screening, and treatment of anemia as a priority in maternal health care, and iron supplementation might optimize infant sleep and decrease the risk of infant sleep disorders. Policies are also needed to reduce the prevalence of anemia during pregnancy, eliminate potential risk factors, and provide support for maternal and child health. Further research is needed to replicate these preliminary findings with objective recordings and to examine the potential association between infant anemia at birth and sleep.

## Data availability statement

The original contributions presented in the study are included in the article/Supplementary material, further inquiries can be directed to the corresponding authors.

## Ethics statement

The studies involving human participants were reviewed and approved by Ethics Committees of Anhui Medical University. The patients/participants provided their written informed consent to participate in this study. Written informed consent was obtained from the individual(s) for the publication of any potentially identifiable images or data included in this article.

## Author contributions

LZ, SM, FD, QL, LW, LY, TX, and PZ: data curation. LZ and SM: formal analysis and writing original draft. LZ, SM, FD, QL, LW, LY, and TX: investigation. LZ, SM, DZ, and PZ: methodology. DZ: project administration. LZ: visualization. All authors contributed to the article and approved the submitted version.

## Funding

This study was funded by National Natural Science Foundation of China (81872631, 82173531), National Key R&D Program of China (2022YFC2702901) and Key Projects of Excellent Young Talents Fund in universities of Anhui Province (gxyqZD2018025).

## Conflict of interest

The authors declare that the research was conducted in the absence of any commercial or financial relationships that could be construed as a potential conflict of interest.

## Publisher’s note

All claims expressed in this article are solely those of the authors and do not necessarily represent those of their affiliated organizations, or those of the publisher, the editors and the reviewers. Any product that may be evaluated in this article, or claim that may be made by its manufacturer, is not guaranteed or endorsed by the publisher.

## Abbreviations

BISQ: brief infant sleep questionnaire
NSD: nocturnal sleep duration
REM: rapid eye movement
SGA: small for gestational age
LGA: large for gestational age
Hb: hemoglobin
BMI: body mass index
SBP: systolic blood pressure
DBP: diastolic blood pressure
IDA: iron deficiency anemia
HPA axis: hypothalamic–pituitary–adrenal axis
