# Supplementary material for: Anemia in pregnancy and sleep of 6-month-old infants: A prospective cohort study
Source: Front Nutr. 2023 Mar 10;10:1049219. doi: 10.3389/fnut.2023.1049219 (PMC10036361; doi:10.3389/fnut.2023.1049219)
Supplement: Supplementary file 1 [file Data_Sheet_1.docx]

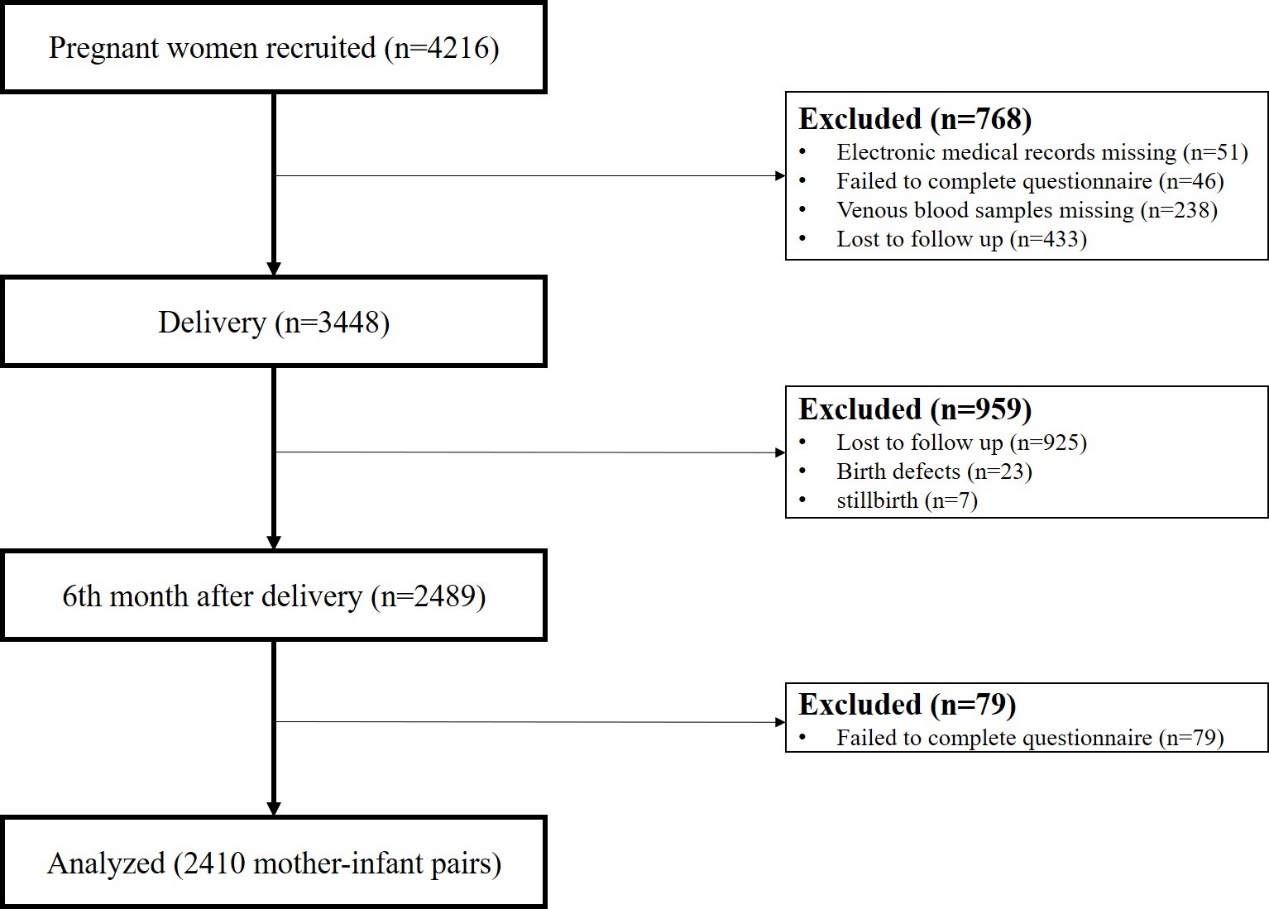


**Supplemental Figure 1 Flow diagram of study participants enrollment, follow-up and analysis.**

**Supplemental table 1 Difference of nocturnal sleep duration in infants born to mothers with anemia vs mothers without anemia (n=2410).**

| **Groups** | **n** | **Anemia** | **None anemia** | ***P* value^a^** |
| --- | --- | --- | --- | --- |
| Overall | 2410 | 560.29±79.57 | 574.27±75.36 | <0.001 |
| Education levels |  |  |  |  |
| >12y | 1618 | 561.57±75.96 | 574.27±75.77 | 0.008 |
| ≤12y | 792 | 557.91±85.97 | 574.25±74.53 | 0.002 |
| Family income |  |  |  |  |
| ≥10000 yuan/mon | 1262 | 559.44±75.42 | 575.06±76.78 | 0.001 |
| <10000 yuan/mon | 1148 | 561.04±83.13 | 573.53±74.05 | 0.009 |
| Pre-pregnancy obesity |  |  |  |  |
| Yes | 421 | 568.79±71.93 | 569.64±74.55 | 0.917 |
| No | 1989 | 558.92±80.7 | 575.5±75.51 | <0.001 |
| Gender |  |  |  |  |
| Male | 1258 | 561.8±71.48 | 573.53±73.93 | 0.008 |
| Female | 1152 | 558.73±87.17 | 575.1±76.98 | 0.002 |
| Preterm birth |  |  |  |  |
| Yes | 225 | 567.07±87.3 | 565.03±74.66 | 0.856 |
| No | 2185 | 559.59±78.77 | 575.22±75.39 | <0.001 |
| SGA |  |  |  |  |
| Yes | 207 | 540.00±81.96 | 569.87±75.03 | 0.015 |
| No | 2203 | 561.74±79.25 | 574.73±75.4 | <0.001 |
| LGA |  |  |  |  |
| Yes | 335 | 556.32±83.19 | 584.77±72.13 | 0.001 |
| No | 2075 | 560.96±78.98 | 572.61±75.75 | 0.001 |
| Breastfeeding |  |  |  |  |
| Yes | 1354 | 563.47±79.88 | 577.2±74.82 | 0.002 |
| No | 1056 | 555.96±79.05 | 570.61±75.92 | 0.004 |

a: p value of covariance analysis. The covariance analysis model adjusted for maternal age at delivery, education levels (if not stratified), family income (if not stratified), pre-pregnancy BMI (if not stratified), SBP, DBP, daily iron supplement, maternal diet (fruits, dessert, vegetables, bean products), infant gender, preterm birth, SGA (if not stratified), LGA (if not stratified), breastfeeding (if not stratified). SGA: small for gestational age; LGA: large for gestational age. BMI: Body mass index; SBP: systolic blood pressure; DBP: diastolic blood pressure; SGA: small for gestational age; LGA: large for gestational age.
